# Environmental DNA Marker Development with Sparse Biological Information: A Case Study on Opossum Shrimp (*Mysis diluviana*)

**DOI:** 10.1371/journal.pone.0161664

**Published:** 2016-08-23

**Authors:** Kellie J. Carim, Kyle R. Christianson, Kevin M. McKelvey, William M. Pate, Douglas B. Silver, Brett M. Johnson, Bill T. Galloway, Michael K. Young, Michael K. Schwartz

**Affiliations:** 1 National Genomics Center for Wildlife and Fish Conservation, U.S. Forest Service Rocky Mountain Research Station, 800 E. Beckwith Ave., Missoula, Montana, 59801, United States of America; 2 Department of Fish, Wildlife and Conservation Biology, Colorado State University, 1474 Campus Delivery, Fort Collins, Colorado, 80523–1474, United States of America; Central Michigan University, UNITED STATES

## Abstract

The spread of *Mysis diluviana*, a small glacial relict crustacean, outside its native range has led to unintended shifts in the composition of native fish communities throughout western North America. As a result, biologists seek accurate methods of determining the presence of *M*. *diluviana*, especially at low densities or during the initial stages of an invasion. Environmental DNA (eDNA) provides one solution for detecting *M*. *diluviana*, but building eDNA markers that are both sensitive and species-specific is challenging when the distribution and taxonomy of closely related non-target taxa are poorly understood, published genetic data are sparse, and tissue samples are difficult to obtain. To address these issues, we developed a pair of independent eDNA markers to increase the likelihood of a positive detection of *M*. *diluviana* when present and reduce the probability of false positive detections from closely related non-target species. Because tissue samples of closely-related and possibly sympatric, non-target taxa could not be obtained, we used synthetic DNA sequences of closely related non-target species to test the specificity of eDNA markers. Both eDNA markers yielded positive detections from five waterbodies where *M*. *diluviana* was known to be present, and no detections in five others where this species was thought to be absent. Daytime samples from varying depths in one waterbody occupied by *M*. *diluviana* demonstrated that samples near the lake bottom produced 5 to more than 300 times as many eDNA copies as samples taken at other depths, but all samples tested positive regardless of depth.

## Introduction

The opossum shrimp *Mysis diluviana* is a small, glacial relict crustaceans native to lakes in the boreal and Laurentian bioregions of North America [1, 2]. *Mysis diluviana* are light-sensitive, coldwater stenotherms (persisting in a narrow temperature range with an optimal temperature of 9°C; [3]) that exhibit diel vertical migration. Opossum shrimp typically inhabit deep water during daylight hours and migrate vertically in the water column at night to feed on zooplankton [4]. Warm summer water temperatures may confine their distribution to only a portion of a water body e.g., the hypoliminion.

Beginning in 1949, *M*. *diluviana* were introduced as prey for sport fish to many lakes and reservoirs in northern North America outside of their native range [5]. These introductions resulted in unintended shifts in community composition resulting in negative effects on native species [6, 7]. Though no longer stocked in North America, *M*. *diluviana* range expansion through water diversions such as the Colorado-Big Thompson Project has been documented [8]. Because of the influence of this species on faunal assemblages in lakes and the possibility for future invasions, reliably detecting this species is a priority for fisheries managers. Sampling of this species most commonly involves vertical tows with plankton nets after dark [9]. Because *Mysis* species are strong swimmers that can avoid nets [10], relatively large (≥ 0.5 m diameter) plankton nets are required to maximize detection probability. Still, low density populations may not be detected by netting, and some waters may not be accessible to boats capable of deploying large plankton nets. More sensitive methods with fewer sampling constraints would be helpful for early detection of invasions and determining the present distribution of this species, and prioritizing areas for management to prevent its further spread.

Environmental DNA (eDNA) sampling is a potential alternative for detection of *M*. *diluviana* because it is a highly sensitive and cost-effective way to detect species that may be rare or difficult to sample [11]. Environmental DNA samples are typically collected by filtering water, and isolating DNA cells off the filter [12]. Because this methods captures free-floating DNA from the environment, the resulting DNA extract contains DNA of many species. Analysis to determine the presence of a particular species from this extract typically requires the use of a species-specific genetic marker, generally a partial sequence of a mitochondrial region that is unique to the taxon of interest. Development of a marker that is applicable across a species' range requires that it amplify all haplotypes of the target species and fails to amplify the DNA of any other taxon, especially of closely related and occasionally sympatric non-target species [13, 14]. The first step of eDNA marker development requires analysis of sequence data to identify areas of the mitochondrial genome that are conserved within a species, but vary between the target and non-target species. Environmental DNA markers that are built in areas of high sequence divergence between target and non-target species are less likely to give false positive results from amplification of non-target DNA. However, because the number and location of base-pair mismatches can influence the performance of eDNA markers, they must be tested against DNA of non-target taxa in the laboratory to confirm species specificity [14]. This approach requires both an extensive library of available sequences for initial screening *in silico*, and the ability to acquire appropriate tissues for confirmation *in vitro*.

For many taxa, however, few or no sequences are available in public databases and tissue samples may be difficult to obtain or rarely archived. For example, a recent paper looking at marine crustaceans noted that of the 17,635 morphologically described Decapoda species (e.g., crayfish, crabs, and lobsters) only 5.4% were represented by cytochrome C oxidase subunit I (COI) sequences [15], despite the fact that this genomic region is the target of a global effort to catalog all taxa [16]. Furthermore, consensus between taxonomies described by morphological versus genetic information may be low [17, 18], and many morphologically described species are indistinguishable using short regions of the mitochondrial genome [19, 20]. Finally, many species groups lack a comprehensive tissue archive suitable for DNA analysis. If published genetic sequence information and tissues for screening markers are scarce, then developing a species-specific marker that can be applied 1) for broad-scale detection of the target species and 2) with specificity to the target species is difficult to demonstrate.

In this paper, we present two novel approaches to develop a species-specific eDNA marker when tissue samples and publically available sequence data target and closely related non-target species are largely unavailable. The first approach entails development of multiple eDNA markers that reliably identify the target, in this case, *M*. *diluviana*. Here, a positive detection at both markers decreases the likelihood of false positive detections resulting from unscreened non-target organisms. The second is the use of existing sequence data to construct synthetic sequences to challenge markers when sample DNA are unavailable, using synthetic DNA as a proxy for tissue-derived DNA.

## Methods

### Environmental DNA marker development

We compiled and aligned sequence data from the cytochrome C oxidase subunit I (COI) region of the mitochondrial genome from GenBank for 29 *M*. *diluviana* individuals collected from sites across North America [1, 21, 22] and 15 individuals from eight other crustacean species commonly found in freshwaters of the western U.S. (Table 1). We used these sequences and the *DECIPHER* package [23] in R v. 3.1.0 [24] to generate two candidate primer sets (Mysis_A and Mysis_B) in the COI region that would be specific to *M*. *diluviana* (Table 2), and screened candidate primers for secondary structures using IDT OligoAnalyzer (https://www.idtdna.com/calc/analyzer).

**Table 1:** A list of species and corresponding GenBank accession number for sequences used in marker development.

Order	Genus	Species	GenBank Accession
Mysidae	*Mysis*	*diluviana*	AY920494.1, DQ189153.1 -DQ189155.1, EF609241.1—EF609265.1
Isopoda	*Asellus*	*aquaticus*	DQ144893.1
Cladocera	*Bosmina*	*coregoni*	AY075057.1
Cladocera	*Daphnia*	*lumholtzi*	KC154293.1
Amphipoda	*Gammarus*	*lacustris*	JX899356.1
Amphipoda	*Hyalella*	*sandra*	DQ464675.1—DQ464682.1
Decapoda	*Orconectes*	*rusticus*	AY701248.1
Decapoda	*Orconectes*	*virilis*	AF474365.1
Decapoda	*Procambarus*	*clarkii*	KJ645855.1

**Table 2:** Primer and probe sequences for the Mysis_A and Mysis_B markers.

Oligo	Sequence	Final concentration (nM)
Mysis_A Forward	5'-CCAGTGTTAGCAGGGGCTAT-3'	600
Mysis_A Reverse	5'-CCCACCTACAGGGTCAAAGA-3'	600
Mysis_A Probe	5'-TTTAACAGACCGTAATTTAA-3'	250
Mysis_B Forward	5'-GAGTTTTAATTCGGTTAGAGTTAGGGC-3'	900
Mysis_B Reverse	5'-CATGCGCAGTAACAATTACGTTATAA-3'	900
Mysis_B Probe	5'-CATTTGATTGGGGACAGACA-3'	250

We obtained tissues of *M*. *diluviana* from Flathead Lake, MT (Flathead County, approximately 47.8999°N -114.1000°W) and Carter Lake, CO (Larimer County, approximately 40.3308°N -105.2178°W) for marker screening. Individuals from each location were preserved in 70% ethanol prior to DNA extraction. DNA was extracted from each sample by freezing specimens using liquid nitrogen, grinding the tissue to a coarse powder and then following the QIAGEN DNeasy Blood and Tissue Kit following the manufacturer’s protocol. We tested the candidate primer sets against a total of six individuals (three from each location) using 4 μl of DNA extracted from tissue, 10 μl of 1X concentration of SYBR Green PCR Mastermix (Life Technologies), 2 μl of each primer at 150 nM concentration, and 2 μl of water. We used cycling conditions of 95°C/10 min [95°C/15 s, 60°C/60 s] × 45 cycles on a StepOne Plus Real-time PCR Instrument (Life Technologies), followed by a melt curve from 65°C to 95°C in 0.3°C increments to test for primer dimer formation.

We designed a hydrolysis probe (TaqMan-MGB) using PrimerExpress 3.0 (Life Technologies) for each primer set (Table 2). We screened candidate primers for secondary structures using IDT OligoAnalyzer (https://www.idtdna.com/calc/analyzer). Each probe had three to four nucleotide mismatches when compared to published sequences of non-targets in the orders Amphipoda and Isopoda (Table 1), where a single nucleotide mismatch in the probe is often sufficient to prevent DNA amplification [13].

We optimized primer concentrations for each complete marker set (i.e., primers and corresponding probe) to increase marker specificity and for ease of future multiplexing following the methods of Wilcox et al. [13], using the same qPCR cycling conditions as for primer testing above, except without a melt curve. The optimal combination of primers was that with the lowest concentration of each primer that resulted in the lowest cycle threshold (C_t_) value yet maintained high end-point fluorescence relative to the highest primer concentration (Table 2).

To estimate the efficiency and precision of both the Mysis_A and Mysis_B markers, we tested marker sensitivity of both Mysis_A and Mysis_B by creating a five-level standard curve dilution series (6 250, 1 250, 250, 50, and 10 copies per 4 μl) for each marker. This standard curve was created by quantifying PCR product (*M*. *diluviana* tissue amplified with each marker) from the above analysis on a Qubit Fluorometer (Thermo Fisher Scientific) and diluting the DNA in TE buffer to the desired concentrations. We ran six replicates of each dilution using optimized primer concentrations for each marker using the same qPCR set up and cycling conditions as for primer optimization above.

### Marker validation

We were unable to obtain tissues of non-target crustacean species likely to overlap with *M*. *diluviana* in western North America due to the incomplete understanding of species diversity and distribution within this group, as well as the paucity of specimens available for analysis. As a result, we did not screen Mysis_A and Mysis_B against DNA of most non-target species. However, we used three alternative approaches to decrease the potential that non-target amplification would occur.

First, to verify that the primers and probes from either set would not amplify DNA from known species with published DNA sequences, we performed a nucleotide BLAST search [25] with the sequences for each primer and the probe. Specifically, we looked for any published sequences of non-target species that are an exact, or near exact match for all three components of the eDNA marker, which could result in non-target amplification. Second, we used synthetic DNA matching sequences data from closely related species that may overlap in range to screen both markers. *Asellus aquaticus* is in the class Malacostraca with *M*. *diluviana*, and is one of the most closely related species known to overlap with *M*. *diluviana* in western North America. We used two synthetic DNA fragments 90 and 98 basepairs in length from the COI gene of *A*. *aquaticus* (GenBank accession DQ144893.1), which included the 78 basepair region of the Mysis_A marker and 84 basepair region of the Mysis_B marker, respectively. While there is currently no known populations in the Rocky Mountains, *Hemimysis anomala* is a crustacean in the Mysidae family that has recently invaded the areas of the Great Lakes in North America [26]. To verify that we would be able to detect *M*. *diluviana* in the presence of this closely related species, we also screened the Mysis_B marker against a synthetic sequence matching 97 basepair fragment of *H*. *anomala* (Genebank accession # EU029164.1). We were unable to create a synthetic DNA fragment for *H*. *anomala* which overlapped with Mysis_A because none of the publically available sequences (GenBank, n = 9) contained sequences data overlapping this segment of the COI gene. Synthetic DNA was obtained by ordering a synthetic gene for each fragment specified above (Integrated DNA Technologies). To prepare the synthetic DNA for screening, the lyophilized gene was resuspended in TE, linearized with a Pvu1 restriction digest and purified using PurLink® APCR Purification Kit (Invitrogen™). The synthetic DNA fragments were screened using the appropriate marker with optimized primer concentrations, and following the same PCR set up and cycling conditions as for primer optimization above.

**Fig 1:**
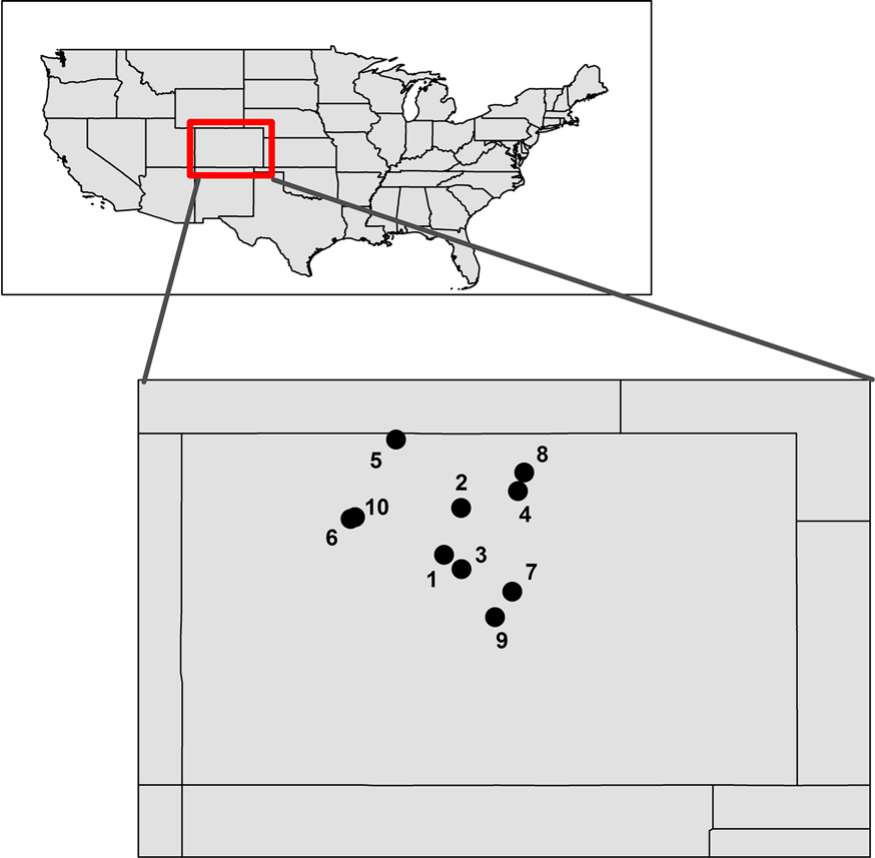
Map of waterbodies sampled for eDNA detection of *M*. *diluviana* in Colorado, USA. Numbers correspond to Map ID in Table 3.

Finally, to verify that both markers performed as expected when applied to field samples, we screened both markers using eDNA samples collected from 10 reservoirs in Colorado (Fig 1; Table 3): five where *M*. *diluviana* were known to be present (Carter Lake, Dillon Reservoir, Lake Granby, Lower Big Creek Lake, and Jefferson Lake), two where *M*. *diluviana* were not introduced and have never been observed (Elevenmile Canyon Reservoir and Upper Stillwater Reservoir), and three where *M*. *diluviana* had been introduced but were not detected with traditional sampling methods in 2014, 2015, and 2016, respectively (Cheesman Reservoir, Stillwater Reservoir, and Horsetooth Reservoir).

**Table 3:** Summary of sample collection and results. Year introduced refers to the earliest year of known *M*. *diluviana* stocking from Nesler et al. (1986). *M*. *diluviana* density (individuals /m^2^) was estimated using traditional sampling methods. Density estimates were taken within 24 hours of eDNA collection. DNA was quantified using both markers and is listed in copies / L of water sampled.

Water body	Year introduced	Density (SD)	eDNA collection date	Sample strata (depth in m)	eDNA sampling coordinates		DNA quantity (SD)	
					Latitude	Longitude	Mysis_A	Mysis_B
Dillon Reservoir	1970	171 (62)	6/18/2014	Bottom (58)	39.614399	-106.061571	13358 (959)	4929 (46)
			6/18/2014	Surface (0)	39.616058	-106.061433	73 (29)	15 (10)
			6/18/2014	Thermocline (10)	39.610441	-106.060858	43 (3)	28 (8)
			6/18/2014	Bottom (50)	39.612698	-106.056373	17410 (407)	4878 (357)
			6/18/2014	Surface (0)	39.611319	-106.060417	107 (39)	22 (8)
			6/18/2014	Thermocline (10)	39.616639	-106.050002	58 (69)	121 (47)
			7/10/2014	Bottom (50)	39.610823	-106.065302	359 (44)	185 (34)
			7/10/2014	Surface (0)	39.610100	-106.066619	9 (0)	3 (0)
			7/10/2014	Thermocline (10)	39.600374	-106.060099	68 (43)	27 (8)
Lake Granby	1971	294 (94)	8/13/2015	Bottom (52)	40.151051	-105.865884	399 (99)	272 (16)
Jefferson Lake	1972	392 (137)	8/11/2015	Bottom (50)	39.454677	-105.861831	82 (24)	44 (27)
Carter Lake	1971	515 (154)	10/7/2015	Bottom (34)	40.343045	-105.218825	1580 (266)	1010 (52)
Lower Big Creek Lake	1969	3 (3)	9/10/2015	Bottom (18)	40.929748	-106.608460	35 (16)	25 (6)
Stillwater Reservoir	1974	0	7/16/2015	Bottom (14)	40.025420	-107.125101	0	0
Cheesman Reservoir	1971	0	6/19/2014	Bottom (50)	39.197614	-105.284399	0	0
			6/19/2014	Bottom (49)	39.202444	-105.284407	0	0
			6/19/2014	Bottom (57)	39.201661	-105.284207	0	0
Horsetooth Reservoir	1971	0	6/3/2016	Bottom (44)	40.553807	-105.149198	0	0
			6/3/2016	Bottom (46)	40.53048	-105.144293	0	0
Eleven Mile Reservoir	Not stocked	0	7/9/2014	Bottom (30)	38.907827	-105.480703	0	0
			7/9/2014	Bottom (30)	38.907601	-105.480863	0	0
			7/9/2014	Bottom (30)	38.907329	-105.481277	0	0
Upper Stillwater Reservoir	Not stocked	0	7/15/2015	Bottom (6)	40.044299	-107.074079	0	0

### Data collection and sample analysis

Each of the 10 reservoirs were sampled for *M*. *diluviana* using a sampling approach which paired eDNA collection with standard methodologies using plankton nets [27]. Net-based sampling was conducted at least 1 h after dark on moonless nights, when *M*. *diluviana* were expected to be distributed in the water column. Three stations (reservoirs < 40.5 ha in surface area), five stations (40.5 ha ≤ surface area < 404.7 acres ha) or 10 stations (reservoirs ≥ 404.7 ha in surface area) were sampled with a 1.0-m diameter, 500-μm mesh conical plankton net towed from 1.0 m above the bottom to the surface at 0.4 m/s with an electric winch. The catch from each haul was preserved in 70% ethanol. Total counts of the catch in each net sample were normalized to individuals/m^2^ based on the cross-sectional area of the net opening (0.785 m^2^)

Environmental DNA samples from the 10 reservoirs were collected during daylight hours from an aluminum research boat following a modified protocol developed by Carim et al. [28]. Water samples were collected 1 m above the bottom of the deepest location in each waterbody (the location in the water column where *M*. *diluviana* were assumed to be most abundant) using a 3-L van Dorn sampler (Wildco Model 1130–045). Water was immediately filtered through a Whatman 47-mm-diameter, 1.5-μm glass microfiber filter (GE HealthCare) using a peristaltic pump (Cole Parmer Masterflex E/S pump). The van Dorn sampler was sterilized between samples by submersion in a 50% bleach solution for 20 minutes. The van Dorn sampler was then removed from the bleach solution, rinsed in deionized water and air-dried, while carefully avoiding contact with any personnel or gear. The boat was decontaminated after sampling each water body by spraying thoroughly with 160°C water and allowing it to air-dry in sunlight.

To evaluate the relative detection of *M*. *diluviana* DNA at different depths within the water column, additional water samples were collected in Dillon Reservoir at the water surface and 1 m below the thermocline. Prior to sampling, a thermal profile was taken with a YSI ProODO. The thermocline was determined to be the depth of maximum vertical temperature difference in the water column. Surface samples were collected by directly pumping 3 L of water from the side of the boat while slowly moving, following the protocol developed by Carim et al. [28]. Samples below the thermocline were collected using the van Dorn sampler as described above.

A control sample was taken prior to each field sample to test for equipment contamination. Controls for surface samples were collected by directly filtering 500 mL of distilled water. Control samples for thermocline and near-bottom samples were collected by filtering 500 mL of distilled water that had been swirled in the van Dorn sampler for approximately 10 s.

All filters were stored in silica desiccant and immediately chilled (approx. 0°C) and then placed in a freezer (-10°C) until being sent to the National Genomics Center for Wildlife and Fish Conservation for analysis. Samples were stored at -20°C at the National Genomics Center for Wildlife and Fish Conservation until DNA extraction occurred. Environmental DNA was extracted from one half of each filter using a QIAGEN DNeasy Blood and Tissue Kit and QIAshredder using a modified protocol [29] with a final elution volume of 100 μl. The second half of each filter was archived at -20°C for future analysis. If more than one filter was used to collect the sample, DNA from one half of each filter was combined after initial lysis incubation in the extraction process. All DNA extracted from environmental samples was stored at -20°C until qPCR analysis occurred.

Samples and paired field control samples were tested against both Mysis_A and Mysis_B in triplicate with an internal positive control (IPC; TaqMan® Exogenous Internal Postive Control Reagents, Thermo Fisher Scientific) to test for the presence of inhibitors limiting PCR amplification. This qPCR analysis occurred in 15-μl reaction volumes containing 7.5 μl Environmental Mastermix 2.0 (Life Technologies), 0.75 μl of 20X assay, 4 μl of DNA extracted from tissue, 1.5 μl 10X IPC Mix, 0.3 μl 50X IPC DNA, and 0.95 μl water using the same cycling conditions as for primer optimization above. Each PCR plate also included a triplicate negative control to screen for contamination in PCR reagents. DNA was quantified for all samples using the Mysis_A marker with the standard curve analysis described above. To quantify eDNA copy numbers in relation to water depth, samples from Dillon Reservoir were amplified using a standard curve dilution series with the same concentrations as above in marker development. The standard curve was run in triplicate on a PCR plate with all Dillon Reservoir samples to estimate DNA concentrations. We computed the average of the 3 reactions associated with each sample and then multiplied this average by 16.67 to estimate quantities per liter of sampled water. (DNA was extracted from half of the filter producing a 100 μl elution volume, each reaction used 4 μl of the elution, and a total of 3 L were filtered. The long version of this calculation is as follows: multiply the average DNA quantity in the triplicate reaction by 25 to estimate the DNA quantity in 100 μl volume of extracted DNA, then multiplied by this number by 2 to estimate all DNA on one entire filter, and then finally divided this number by 3, the total number of liters sampled to reach the estimated number of DNA copies per liter).

To ensure amplification of DNA was not the result of non-target amplification, PCR products from one surface, one thermocline and one bottom sample in Dillion Reservoir (the first three samples listed in Table 3) were sequenced and verified to match *M*. *diluviana* DNA following methods outlined by Valentin et al. [30].

All water sampling occurred on public waterbodies where no permission for collecting water samples or invertebrate specimens were necessary. No endangered species were involved in this research.

## Results

### Marker validation and detection of *M*. *diluviana* eDNA

Both Mysis_A and Mysis_B were invariant across all reference *M*. *diluviana* sequences, and both markers amplified tissue-derived samples efficiently and with high precision (amplification efficiencies were 96.2% and 97.1% with precisions of *r*^*2*^ = 0.995, and *r*^*2*^ = 0.996 for Mysis_A and Mysis_B, respectively). Standard curve analyses indicated that both markers were able to detect *M*. *diluviana* DNA at concentrations of ≤10 copies/4 μl of extract DNA. There were 3–11 mismatches between any of the primers in either primer set and published sequences of species in the orders Amphipoda and Isopoda, the most closely related taxa to *M*. *diluviana* found in freshwaters of North America. Nucleotide BLAST searches identified no non-target species that with exact or near exact matches to both primers and the probe for either marker. Furthermore, neither marker amplified the corresponding *A*. *aquaticus* synthetic DNA sample, and Mysis_B did not amplify the *H*. *anomala* synthetic DNA sample.

Finally, screening of both markers resulted in positive detections of *M*. *diluviana* in all eDNA samples from all five waterbodies known to harbor this species. Sequencing results form a subset of samples in Dillon Reservoir confirmed that the DNA amplicons originated from *M*. *diluviana*. There was no *M*. *diluviana* DNA detected by either marker in samples from the five waterbodies where this species was not captured during netting (Table 3).

For all analyses, all field and laboratory controls were negative for the presence of *M*. *diluviana* DNA, indicating that field sterilization procedures were adequate and that contamination was not a factor affecting results in this study.

### Optimization of field sampling

The amount of *M*. *diluviana* DNA observed in samples obtained near the benthic zone in Dillon Reservoir averaged roughly 10 400 copies per liter of water filtered, compared to an average of roughly 60 copies per liter for surface and thermocline samples (Table 3). Results from the Mysis_B marker followed a similar pattern with an average of over 3 000 copies per liter of water in samples collected near the bottom of the reservoir, compared to an average of 13 and 26 copies per liter for surface and thermocline samples respectively. The quantity of DNA obtained from samples within each stratum was relatively consistent, with the exception of one benthic sample collected on July 10th which had substantially lower DNA quantity relative to other benthic samples (Table 3).

## Discussion

In this study, we successfully developed and implemented eDNA markers for the detection of *M*. *diluviana* in the absence of substantial access to non-target tissues, and also identified sampling techniques to improve efficiency of *M*. *diluviana* detection when collecting field samples. To our knowledge, this is the first study utilizing multiple eDNA markers and synthetic DNA to overcome limitations of sampling non-targets for marker development and validation. We believe that the use of these approaches will aid in development of markers that are robust against false positive detections given relatively limited knowledge of the genetic makeup of non-target organisms that are likely to be present in eDNA samples.

Although we used synthetic DNA in a limited fashion, we believe that this approach holds promise for rapidly testing both a marker’s generality and specificity. The growth of searchable sequence databases has led to a growing gap between *in silico* availability of sequence data at commonly sequenced regions such as COI and maintained and publically available tissue archives. The use of synthetic DNA fragments based on publically available sequence data broadens our ability to both screen and challenge potential markers.

However, it is worth noting that publically available sequence data are not perfect- reported sequences may be incomplete or contain errors, and publically available sequences may not represent all haplotypes of a species, or even all non-target species of interest. Furthermore, synthetic DNA is limited in that it does not contain the entire genome of the non-target species which may have added complexities affecting amplification. We faced these very challenges while attempting to screen both markers against synthetic DNA of *H*. *anomala*. Publically available sequence data for the COI region of this species was incomplete, preventing us from obtaining a synthetic DNA fragment that overlapped with the Mysis_A marker. However, other components of our marker development and testing structure overcome this limitation. Firstly, to validate the presence of *M*. *diluviana*, the testing structure presented here requires a positive detection at both the Mysis_A and Mysis_B markers. Screening of synthetic DNA indicates that the presences of *H*. *anomala* would not result in a positive detection in the Mysis_B marker. As a result, the presence of *H*. *anomala* would not result in false positive detections with our current testing structure, even if there was unintended amplification of this non-target with Mysis_A. Secondly, the screening of negative control field samples from waterbodies where *M*. *diluviana* are absent, but a variety of non-targets organisms are present further tests the likelihood of false positive detections. Specifically, the use of negative control samples allows us to screen for non-target samples that may result in positive detections at one or both markers when the target species is known to be absent. In summary, each of these strategies alone carries assumptions and limitations with regards to accurate eDNA based detections. However, simultaneously using these strategies creates a stronger and more robust assessment, particularly when comprehensive tissue libraries and sequences databases are not available.

The field sampling in Dillon Reservoir indicated that deep-water sampling will provide the most reliable eDNA samples for *M*. *diluviana* in diurnal samples taken during the summer months. While *M*. *diluviana* DNA was detected in all samples collected in Dillon Reservoir, samples collected just above the bottom yielded over 50-fold more DNA compared to samples collected at the surface and below the thermocline in the same reservoir. This is not surprising given that *M*. *diluviana* are most commonly found in deeper waters during daylight [3]. Similar results were observed by Echmiller et al. [31] with common carp (*Cyrpinus carpio*), where higher concentration of carp DNA were obtained in samples collected in areas with higher carp use within in a small lake. For a species like *M*. *diluviana*, samples collected closer to the water’s surface may retain DNA for shorter period of time because shallower depths experience higher temperatures and more UV radiation, factors known to degrade eDNA suspended in water [32].

While one benthic sample produced much lower copy numbers (359 copies/L with Mysis_A; 185 copies/L with Mysis_B) than the other two benthic samples, field notes indicate that a substantial amount of sediment was present in this water sample and that the van Dorn sampler likely hit the bottom of the reservoir. There was no evidence of PCR inhibition in this sample, indicating the presence of sediment did not prevent amplification of existing DNA, but this does suggest that samples impacting the benthos should be avoided when collecting eDNA samples for *M*. *diluviana*. Nevertheless, the quantity of DNA obtained in this sample was on average at least one order of magnitude higher than that observed in any surface or thermocline samples.

Interestingly, the Mysis_A marker generally detected higher DNA quantities than the Mysis_B marker, despite the fact that DNA quantities were obtained with a standard curves using the same DNA concentrations. Estimates between markers were generally within 2 standard deviations of one another, with the exception of samples collected near the benthic zone in Dillon Reservoir, in which Mysis_A detected significantly higher copy number of DNA compared to Mysis_B. While there is no obvious explanation for this variation between markers, the relative DNA amounts obtained for each sample were consistent across markers. That is, samples that had relatively higher DNA concentrations with Mysis_A, also had relatively higher DNA concentrations with Mysis_B. More broadly, we believe that this inconsistency in DNA quantification between markers points to a bias in the ability of qPCR to accurately estimate absolute DNA quantity in a sample. Specifically, while qPCR based methods tend to be more sensitive to detection of targets, they are often not as consistent in estimates of DNA quantity, compared to methods such as drop digital PCR, which directly quantifies DNA copy number without the use of a standard curve, and tend to be less sensitive to the presence of inhibitors [33, 34].

Despite these differences between markers, standard curves analysis indicates that both markers should be able to reliably detect *M*. *diluviana* when there are ≥10 DNA copies per reaction (corresponding to 167 copies per liter). This high level of sensitivity was affirmed in field samples from Dillion Reservoir where both markers successfully detected DNA in samples with estimates of fewer than 10 copies per liter of water. Additionally, eDNA sampling confirmed *M*. *diluviana* presence in Lower Big Creek Lake where traditional sampling methods estimated *M*. *diluviana* density as low as 3 individuals∙m^2^ (Table 3). However, while eDNA samples were less time consuming and logistically easier to obtain these data do not fully elucidate how eDNA copy number directly relates to densities of *M*. *diluviana*, nor they strongly identify one method as more effective at species detection over another. Future studies that directly compare *M*. *diluviana* counts with eDNA copy numbers will be useful in understanding how these methods compare in terms of sensitivity to detection.

In conclusion, this study demonstrates a robust approach to eDNA marker development for organisms when tissue samples of non-target organisms may be difficult to obtain, and when publically available sequence data is sparse. By demanding amplification of target DNA at multiple eDNA markers, we also highlight a robust structure for eDNA based species detection that reduces the risk of false positive detections, particularly when the range sympatric non-target organism may be broad and not fully identified.
