# Correction: Environmental DNA Marker Development with Sparse Biological Information: A Case Study on Opossum Shrimp (*Mysis diluviana*)

**DOI:** 10.1371/journal.pone.0165573

**Published:** 2016-10-21

**Authors:** Kellie J. Carim, Kyle R. Christianson, Kevin M. McKelvey, William M. Pate, Douglas B. Silver, Brett M. Johnson, Benjamin T. Galloway, Michael K. Young, Michael K. Schwartz

The seventh author’s name is spelled incorrectly. The correct name is: Benjamin T. Galloway.
